# Supplementary material for: Nonparametric time series summary statistics for high-frequency accelerometry data from individuals with advanced dementia
Source: PLoS One. 2020 Sep 25;15(9):e0239368. doi: 10.1371/journal.pone.0239368 (PMC7518630; doi:10.1371/journal.pone.0239368)
Supplement: S1 Table — Key demographic characteristics of the 32 resident participants from the study of [33]. The 26 participants with advanced dementia from which we have valid accelerometry data are all from this larger study, where the demographic breakdown between those participants with and without valid accelerometry data is not available. (PDF) [file pone.0239368.s001.pdf]

S1 Table. Demographic variables. Key demographic characteristics of the 32 resident participants from the study of [33]. The 26 participants with advanced dementia from which we have valid accelerometry data are all from this larger study, where the demographic breakdown between those participants with and without valid accelerometry data is not available.

Characteristic	Non-intervention	Intervention
**Sex, n (%)**		
Male	9 (64.3)	8 (44.4)
Female	5 (35.7)	10 (55.6)
**Age (years), mean±SD**	84.7±6.4	79.0±10.5
**Marital status, n (%)**		
Married	11 (78.6)	7 (38.9)
Single	0	3 (16.7)
Widowed	3 (21.4)	8 (44.4)
**Ethnicity, n (%)**		
Black African Caribbean	0	1 (5.6)
White	14 (100.0)	17 (94.4)
**Dementia diagnosis, n (%)**		
Alzheimer's disease	7 (50.0)	7 (38.9)
Vascular dementia	1 (7.1)	5 (27.8)
Dementia with Lewy bodies	2 (14.3)	0
Unspecified dementia	4 (28.6)	6 (33.3)
**Length of stay (years), mean±SD**	2.9±2.6	2.1±1.4
