# Supplementary material for: Nonparametric time series summary statistics for high-frequency accelerometry data from individuals with advanced dementia
Source: PLoS One. 2020 Sep 25;15(9):e0239368. doi: 10.1371/journal.pone.0239368 (PMC7518630; doi:10.1371/journal.pone.0239368)
Supplement: S2 Table — Mean, standard deviation (SD), minimum and maximum values of all statistical measures used in this paper for different groups of participants. The dementia group refers to the combined non-intervention and intervention groups. (PDF) [file pone.0239368.s002.pdf]

S2 Table. Summary statistics. Mean, standard deviation (SD), minimum and maximum values of all statistical measures used in this paper for different groups of participants. The dementia group refers to the combined non-intervention and intervention groups.

Statistical measures	Groups	Mean	SD	Min.	Max.
IS	Non-intervention	0.029	0.019	0.003	0.063
	Intervention	0.039	0.025	0.005	0.082
	Dementia	0.035	0.023	0.003	0.082
	Without Dementia	0.147	0.054	0.062	0.257
IV	Non-intervention	1.605	0.137	1.424	1.856
	Intervention	1.519	0.186	1.036	1.775
	Dementia	1.555	0.169	1.036	1.856
	Without Dementia	1.028	0.301	0.376	1.487
α (overall)	Non-intervention	0.816	0.057	0.722	0.946
	Intervention	0.878	0.050	0.808	0.965
	Dementia	0.852	0.061	0.722	0.965
	Without Dementia	1.005	0.098	0.843	1.174
α (daytime)	Non-intervention	0.820	0.066	0.717	0.983
	Intervention	0.875	0.053	0.792	0.962
	Dementia	0.852	0.064	0.717	0.983
	Without Dementia	1.006	0.097	0.842	1.175
α (nighttime)	Non-intervention	0.806	0.067	0.710	0.891
	Intervention	0.897	0.053	0.825	1.004
	Dementia	0.859	0.074	0.710	1.004
	Without Dementia	0.905	0.083	0.741	1.043
$\mathrm{PoV}^{(F)}$	Non-intervention	0.027	0.031	0.001	0.101
	Intervention	0.049	0.046	0.002	0.138
	Dementia	0.040	0.041	0.001	0.138
	Without Dementia	0.152	0.065	0.077	0.305
$\mathrm{PoV}^{(H)}$	Non-intervention	0.055	0.036	0.007	0.122
	Intervention	0.078	0.052	0.012	0.167
	Dementia	0.068	0.047	0.007	0.167
	Without Dementia	0.224	0.071	0.108	0.338
