# Supplementary figures and images for: Nonparametric time series summary statistics for high-frequency accelerometry data from individuals with advanced dementia
Source: PLoS One. 2020 Sep 25;15(9):e0239368. doi: 10.1371/journal.pone.0239368 (PMC7518630; doi:10.1371/journal.pone.0239368)

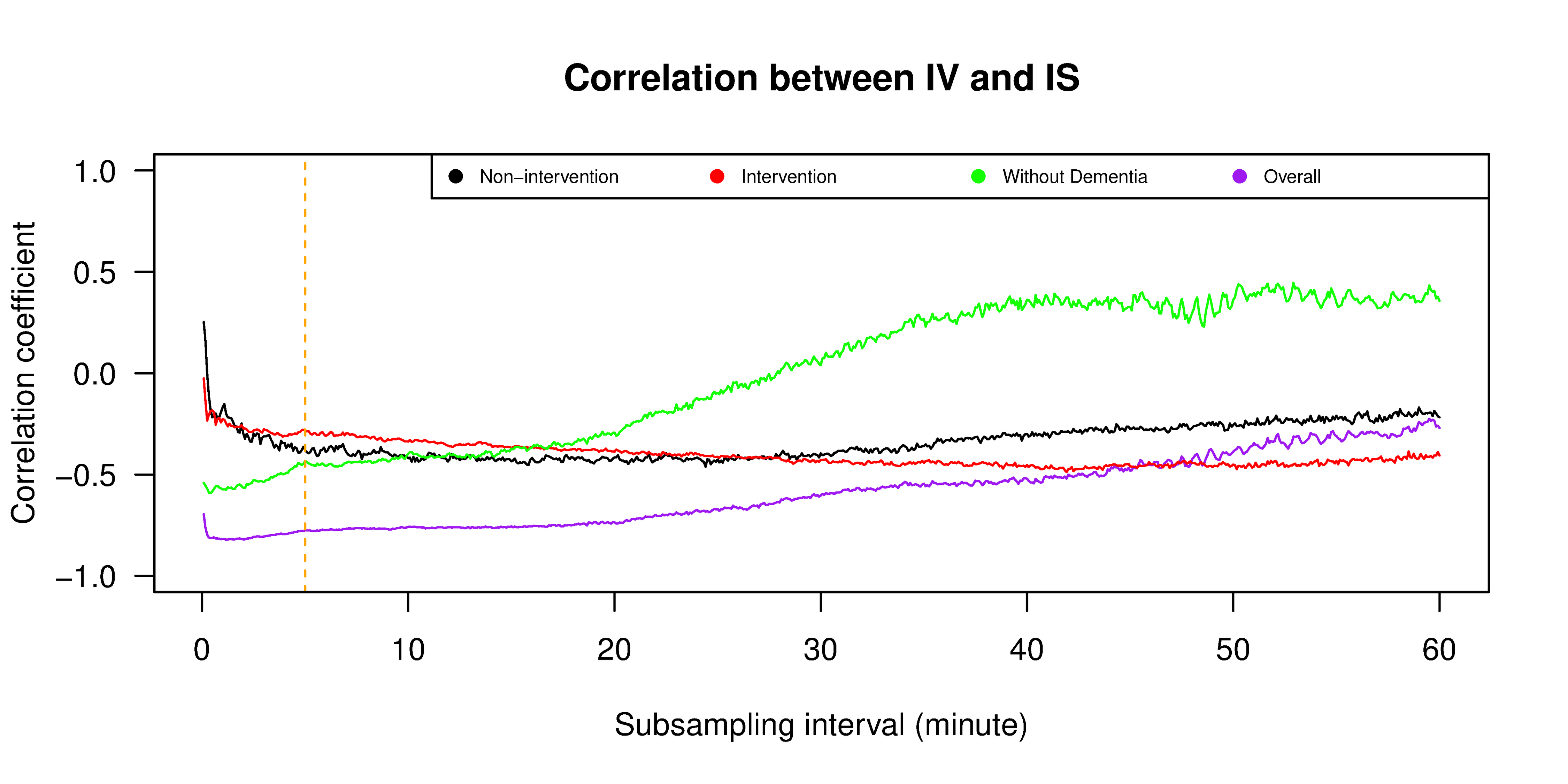

Supplement: S1 Fig — Line plots showing Pearson’s correlation coefficients between IV and IS for the ENMO data with subsampling interval for calculating IV varied from 5 seconds to 60 minutes. The non-intervention group, the intervention group, the group of individuals without dementia, and the overall group are presented by black, red, green, and purple lines, respectively. The orange dashed line refers to 5 minutes and indicates our recommended subsampling interval for calculating IV. (TIF) [file pone.0239368.s003.tif]

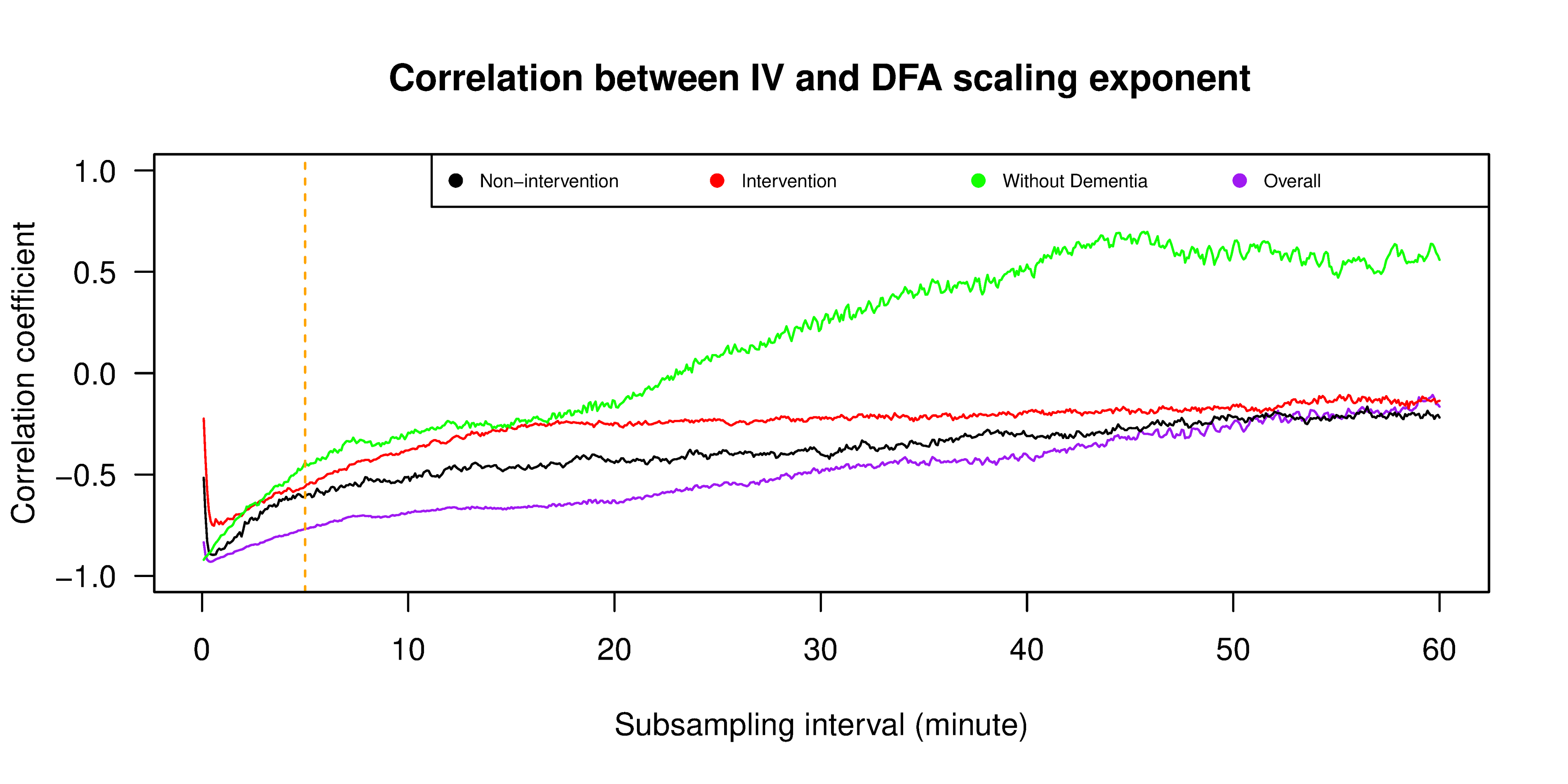

Supplement: S2 Fig — Line plots showing Pearson’s correlation coefficients between IV and the DFA scaling exponent for the ENMO data with subsampling interval for calculating IV varied from 5 seconds to 60 minutes. The non-intervention group, the intervention group, the group of individuals without dementia, and the overall group are presented by black, red, green, and purple lines, respectively. The orange dashed line refers to 5 minutes and indicates our recommended subsampling interval for calculating IV. (TIF) [file pone.0239368.s004.tif]

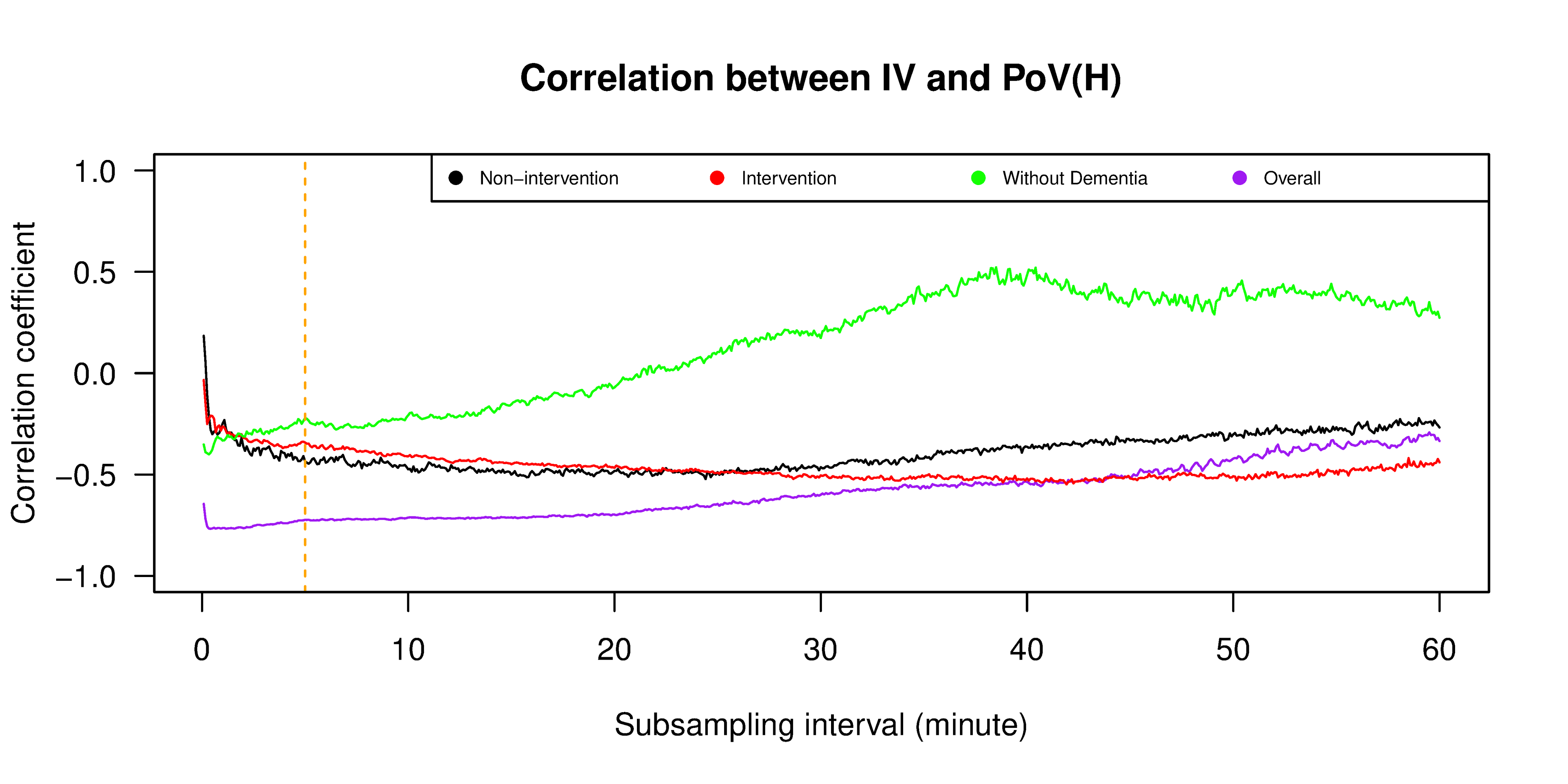

Supplement: S3 Fig — Line plots showing Pearson’s correlation coefficients between IV and PoV around the first four harmonic frequencies for the ENMO data with subsampling interval for calculating IV varied from 5 seconds to 60 minutes. The non-intervention group, the intervention group, the group of individuals without dementia, and the overall group are presented by black, red, green, and purple lines, respectively. The orange dashed line refers to 5 minutes and indicates our recommended subsampling interval for calculating IV. (TIF) [file pone.0239368.s005.tif]
