# Supplementary material for: Nonparametric time series summary statistics for high-frequency accelerometry data from individuals with advanced dementia
Source: PLoS One. 2020 Sep 25;15(9):e0239368. doi: 10.1371/journal.pone.0239368 (PMC7518630; doi:10.1371/journal.pone.0239368)
Supplement: Attachment — Submitted filename: Response to Reviewers.pdf [file pone.0239368.s008.pdf]

RESPONSE LETTER

Thank you for your comments and inviting us to submit a revised version of our manuscript. We would like to thank both reviewers for their expertise and insights into our research. We believe we have addressed all three interesting questions and concerns the first reviewer has raised. We provide our responses in the usual point-by-point format below, where we highlight the changes we have made in the manuscript to reflect their comments.

Reviewer #1

Comment #1: The study compares 3 populations: i) non-intervention group and ii) intervention group, for which demographic data is available in S1_Table, and iii) group without dementia that corresponds to members of the research team and colleagues, and for which no demographic data is available. Groups i) and ii) are elderly adults with an average age of 79-85yo, whereas group iii) are active young or mature adults. Is it correct to compare groups i) and ii) on the one hand and group iii) on the other hand? If so, what does this comparison teach us? Clearly differences are not only about the presence or absence of dementia. The conclusion section should be redacted accordingly.

Response: Thank you for pointing this out, and we agree that the precise wording of the conclusions required some modification. Please find the new wording in the Conclusion of the revised manuscript (page 16 now reads "Limitations of our data analysis include having a relatively small number of participants, and that the without dementia group data comes from individuals from the research group with younger ages..."), where we have made clear the limitation that the without dementia group has a different demographic profile, meaning that we cannot conclude that the difference of statistical measures between participants is solely due to the condition of have advanced dementia or not.

Comment #2: The manuscript refers both to "actigraphy" and "accelerometry". The data used in the present study was accelerometry, whereas most of the previous studies on which the present study builds used actigraphy. It might be useful to explain in the introduction the difference between both types of data, which may not be clear for all readers.

Response: Thank you again for pointing out this important distinction. We have now been consistent and clear on this point in the revised manuscript. As such we have modified the first few lines of the Introduction (Page 1 now reads "Accelerometry data provides a method for monitoring physical activity over time...") to make clear that we work with accelerometry data, and then in the Materials and Methods Section we make clear the precise difference between ENMO/accelerometry data and actigraphy data such as counts per time interval (Page 4 now reads "We emphasise that in the literature other forms of actigraphy time series are sometimes studied...").

Comment #3: Do the authors think that the "U" shape of IV as a function of the sampling frequency in refs. [6,9] and the different shape of their IV curve may be due to the fact that refs. [6.9] were using actigraphy data whereas in the present manuscript accelerometry data was used? Clearly both types of data will have different kinds of autocorrelation properties.

Response: We agree that the differences here may be due to the difference in type of accelerometry/actigraphy data studied. As such we have acknowledged this possibility in the discussion (Page 9 now reads "We note however that these studies were on a range of simulated data, as well as human and animal participants, and used different actigraphy output by studying the counts per time interval rather than ENMO accelerometry data..."). We hope that this analysis is repeated in future experiments to shed further light on the interesting question of optimal subsampling for calculating IV.

Thank you once again for sharing your expertise and your thorough reading of the paper, which we feel that our manuscript has significantly been improved after incorporating your suggested revisions.
