# Supplementary material for: Nonparametric time series summary statistics for high-frequency accelerometry data from individuals with advanced dementia
Source: PLoS One. 2020 Sep 25;15(9):e0239368. doi: 10.1371/journal.pone.0239368 (PMC7518630; doi:10.1371/journal.pone.0239368)
Supplement: Attachment — Submitted filename: 2020-05-06_referee report.docx [file pone.0239368.s006.docx]

The research problem is to quantify the circadian cycles of populations with and without dementia as a proxy for health. The working hypothesis is that cosinor analysis, which is the standard parametric analysis for circadian rhythms, needs specific cycles of prespecified periods (e.g., 24h) to be present in the data in order to work, and that non-parametric methods are better suited to study this type of data, such as the existing IV, IS and Hurst fractal scaling analysis, and the newly proposed PoV. The authors find that all methods distinguish between the groups with and the group without dementia, and that additionally the Hurst exponent distinguishes between the dementia groups with and without extra treatment.

The research problem is valid, the authors apply all methods adequately, the results obtained are interesting and the paper reads well and is well presented. The focus of the article is more on the statistical analysis than on the physiological interpretation of the numerical results and it would be interesting if the authors could elaborate a little more on the how to interpret the IS, IV, Hurst and PoV values for the different populations. I would recommend publication in PLoS ONE after they solve the following minor problems:

- In clinical research, the name “control group” usually indicates the group of healthy or asymptomatic individuals to be compared to groups of individuals with pathologies. Here, “control group” is used for the individuals with dementia but without additional treatment, compared to a group of individuals with dementia and additional treatment, and a group of individuals without dementia, which is confusing. I understand this name “control group” comes from the previous publications of the authors, refs. [31,32], where only the 2 groups with dementia were studied and that the 3^rd^ group without dementia was added for this article. If the authors do not want to use the name “control group” for the asymptomatic individuals in order to avoid conflict with their previous publications, would it be possible to avoid the name “control” group altogether?

- Are all participants of all 3 groups permanent residents of nursing homes? Are all participants similar with respect to demographic, anthropometric, etc., variables? Could the authors add a table to the main text where group-average values and standard deviations for these variables are compared for all groups, together with p-values to indicate that the only difference between the groups is the condition of dementia and/or the additional treatment?

- I agree with the authors that cosinor might not be the optimal method to analyze circadian cycles in irregular data such as in the case of dementia, but could the authors estimate just how much better non-parametric methods are with respect to a parametric method such as cosinor? I guess that the amplitude of cosinor analysis should be proportional to the power of the fundamental of the spectral analysis, i.e., the measure PoV(F) of the authors? If this is the case, just how much better can the different populations be distinguished by including harmonics as in PoV(H) and/or considering other non-parametric measures such as IS, IV and the Hurst exponent. Can the authors quantitatively confirm that their working hypothesis is correct that the proposed non-parametric measures are better suited to analyze their data?

- I think the data the authors are using should be called “accelerometry” instead of “actigraphy”. Actigraphy is coarse-grained accelerometry according to several different conventions including time above threshold, zero crossings or digital integration (see Ancoli-Israel et al., 2003, Sleep 26: 342), such that the units of actigraphy are usually not the gravity constant g, as in the present article. Ref. [33] which explains the ENMO data used here indeed says, “acceleration signal” and never mentions “actigraphy”. The use of accelerometry instead of actigraphy might actually have helped the authors to realize a separate daytime and night-time analysis with DFA, whereas in the case of actigraphy night-time analysis often becomes difficult because of time series which are mostly zero.

- This is the first time I see an article where Hurst and DFA are treated as synonyms. The two methods are indeed very much related, but I would avoid saying they are the same. The result of DFA analysis is actually the alpha exponent, which varies in a different range than H, with 0<=H<1 and 0<alpha<1.5, and mathematical formulae exist to convert the values of one analysis to values of the other analysis. The explanation on p. 6/18 that 0<H<1 (stationary noise) and 1<H2 (non-stationary walk) seems wrong, the range is always 0<=H<1, both for noises and for walks but with different interpretation, see e.g., Eke et al, 2000, Eur. J. Physiol. 439: 403 and Halley et al., 2004, Fluct Noise Lett. 4: R1.

- A related comment, same page p. 6/18, I have never heard about a threshold alpha=1.2, if true then a reference should be added. According to Eke et al (2000), unlike Hurst analysis which must be applied differently to noises (fGn) and to walks (fBm), DFA may be applied to both without distinction. However, some authors have indeed argued that in the cases of walks better results may be obtained without previous integrating the time series before applying DFA (see e.g., Colas et al., 2019, PLoS ONE 14: e0225817), but the explanation here to first differentiate the data (step 5), then to integrate (step 1) and then to add 1 to the slope, seems redundant.

- p. 3/18, the phrase “…the degree of disrupted fractal regulation was strongly negatively correlated with…” is very difficult to understand.

- p. 6/18 how arbitrary are exactly the daytime (06:00-21:00) and night-time (21:00-06:00) intervals? How were these intervals chosen? Do the nursing homes manage fixed lights on and lights out times?

- p. 7/18 If the data is sampled at 1/5 Hz, then the Nyquist frequency should be 2/5 Hz and not 1/10 Hz as stated in the text.

- p. 8/18 and Fig. 1, in the plot of the 24h average of the participant with dementia with additional treatment, why does the circadian cycle seem to be inverted with more activity during the night than during the day?

- p. 9/18 and Fig. 3 on IV as a function of subsampling interval, the curve has a minimum for small subsampling intervals and converges to a constant for large intervals, but this is rather the opposite behavior from your refs. [6] and [9] where a minimum is obtained for intermediate sample intervals and maxima for both small and large intervals. How do you explain this? In particular, how can there be “a dip in intradaily variability (IV) at small sample intervals where there is high-frequency variability in the data” as stated in the text, whereas intuition would suggest a peak?

- Same page, same figure, refs. [6] and [9] suggest to study the whole range of sampling intervals to analyze IV and to interpret physically/physiologically the time scales where differences between populations maximize. What is the physical/physiological meaning of the specific scale of 5min you focus on? Do you think that in other studies, with other populations, other pathologies, and other measurement equipment also a specific scale of 5min will be found, or will this depend on the particular study?

- p. 9/18 and Figs. S1-S3, can you add a legend to explain the colour coding? If the coding is similar to the previous figures, then the meaning of the red, black and green curve is clear, but this is the first time the colour blue is used?

- Same page, same figures. I agree with the argument of the anticorrelation between IV on the one hand and IS or PoV on the other hand. I find the relation with Hurst less clear, why should there be an anticorrelation between IV and Hurst? I do not think Figs. S1-S3 confirm the statement of the specific scale of 5min, because the blue, red and black curves show anticorrelations for all subsampling intervals. Only the green curve passes from anticorrelation to correlation, but at a scale of 30min and not 5min.

- p. 9/18 and Figs. 2, 4 and 6 what is the physical/physiological interpretation of the larger spread in the boxes of IV, IS and DFA exponent for the participants without dementia? I do not see a larger overlap between the boxes of the populations with insomnia for IV than for IS.

- p. 10/18 and Fig. 5, the range of time scales from which the DFA exponent is derived seems rather small, between 2^4=16 and 2^8=256 time units? Are these time units equal to 5s? Then these time scales correspond to 80-1,280s or 1.3-21min, that seems small to characterize daytime duration of 15h or night-time duration of 9h. How do these curves look like at larger time scales, are they linear or might there be crossover effects as in ref. [6]?

- p- 10/18 and Figs. 6&7, I guess that what the authors want to say with “…for individuals without insomnia their data is a combination of stationary and non-stationary time series…”, is that for these individuals their time series behave more as the mysterious 1/f noise, which is indeed the border between stationary noise and non-stationary walks (see Halley et al., 2004; Eke et al., 2000), and which is empirically found in many physiological time series (e.g., heart rate, actigraphy, EEG, etc.) of young and healthy individuals without anyone really understanding why, see e.g. [6], whereas that of the individuals with dementia goes more towards a non-correlated white noise.

- p. 11/18 and Table 1. Why show in the main text a numerical table for the daytime and night-time Hurst exponent and show the numerical results on IS, IV, overall Hurst and PoV(H) in the supplementary information? Perhaps the 2 tables can be joined and showed in the supplementary information?

- p. 11/18 and Fig. 8. The text says that the fundamental and harmonics of the subject with dementia who receives treatment have much smaller powers than the control subject. This is not clear from a first glance of the figure, only when one focusses on the scales of the figures. Could the authors add a 2^nd^ column with the same spectra but where the vertical scale is maintained constant, to facilitate comparison? Also, the units of the powers are not clear, are these percentages or fractions, do in all cases all power sum up to a total of 1 or 100%?
